# Epithelial MHC Class II Expression and Its Role in Antigen Presentation in the Gastrointestinal and Respiratory Tracts

**DOI:** 10.3389/fimmu.2018.02144

**Published:** 2018-09-25

**Authors:** Jonathan E. Wosen, Dhriti Mukhopadhyay, Claudia Macaubas, Elizabeth D. Mellins

**Affiliations:** Program in Immunology, Department of Pediatrics, Stanford University, Stanford, CA, United States

**Keywords:** epithelial cells, MHC class II, antigen presentation, intestine, respiratory

## Abstract

As the primary barrier between an organism and its environment, epithelial cells are well-positioned to regulate tolerance while preserving immunity against pathogens. Class II major histocompatibility complex molecules (MHC class II) are highly expressed on the surface of epithelial cells (ECs) in both the lung and intestine, although the functional consequences of this expression are not fully understood. Here, we summarize current information regarding the interactions that regulate the expression of EC MHC class II in health and disease. We then evaluate the potential role of EC as non-professional antigen presenting cells. Finally, we explore future areas of study and the potential contribution of epithelial surfaces to gut-lung crosstalk.

## Introduction

The epithelium serves as both a physical and chemical barrier as well as an absorptive surface. Our understanding of the function of the aerodigestive epithelium has gradually evolved from its role as a static barrier to a dynamic structure regulating multiple processes. In fact, ECs may have primary immune functions that affect the balance between tolerance and inflammation, as evidenced by expression of MHC class II on the EC surface, an area of exploration several decades ago. Recent evidence suggests there may also be communication with the lung from the gut directed by its microbiome. Because host-microbial interactions first occur at the epithelial surface, re-visiting the role of ECs in antigen processing and presentation is timely. This review aims to synthesize current findings on MHC class II expression in the gut and the lung, explore the role of ECs as non-professional antigen presenting cells (APCs) and discuss how this area may be further investigated as a target for potential diagnostic or therapeutic interventions.

### Structure and function of the aerodigestive epithelium

Though the digestive and respiratory systems are thought of as two entirely distinct anatomic cavities, they have a shared developmental origin from the primitive gut (1, 2). The digestive tube initially extends through the length of the body from which the respiratory tube outpouches, sharing a common embryonic chamber called the pharynx. The linings of these primitive tubes are comprised of embryonic endoderm. The digestive tube eventually differentiates into the components of the gastrointestinal tract including esophagus, stomach, small intestine and colon, due to the interaction of endodermal epithelium with regionally specific mesodermal mesenchyme. The respiratory tube bifurcates into two lungs, with laryngotracheal endoderm becoming the epithelial lining of the trachea, bronchi and lung parenchyma, similarly directed by the regional mesenchyme (3).

By birth, the terminal portion of the digestive tract has matured into the small intestine and large intestine. The small intestine is ~5 m in length and composed of three separate segments—duodenum, jejunum, and ileum—that are exposed to dietary antigens and crucial for oral tolerance. Small intestinal epithelium is composed of finger-like projections called villi and invaginations called crypts of Lieberkühn. The large intestine, or colon, comprises the distal 1.5 m of the gastrointestinal tract and histologically lacks villi (4).

**Figure 1:**
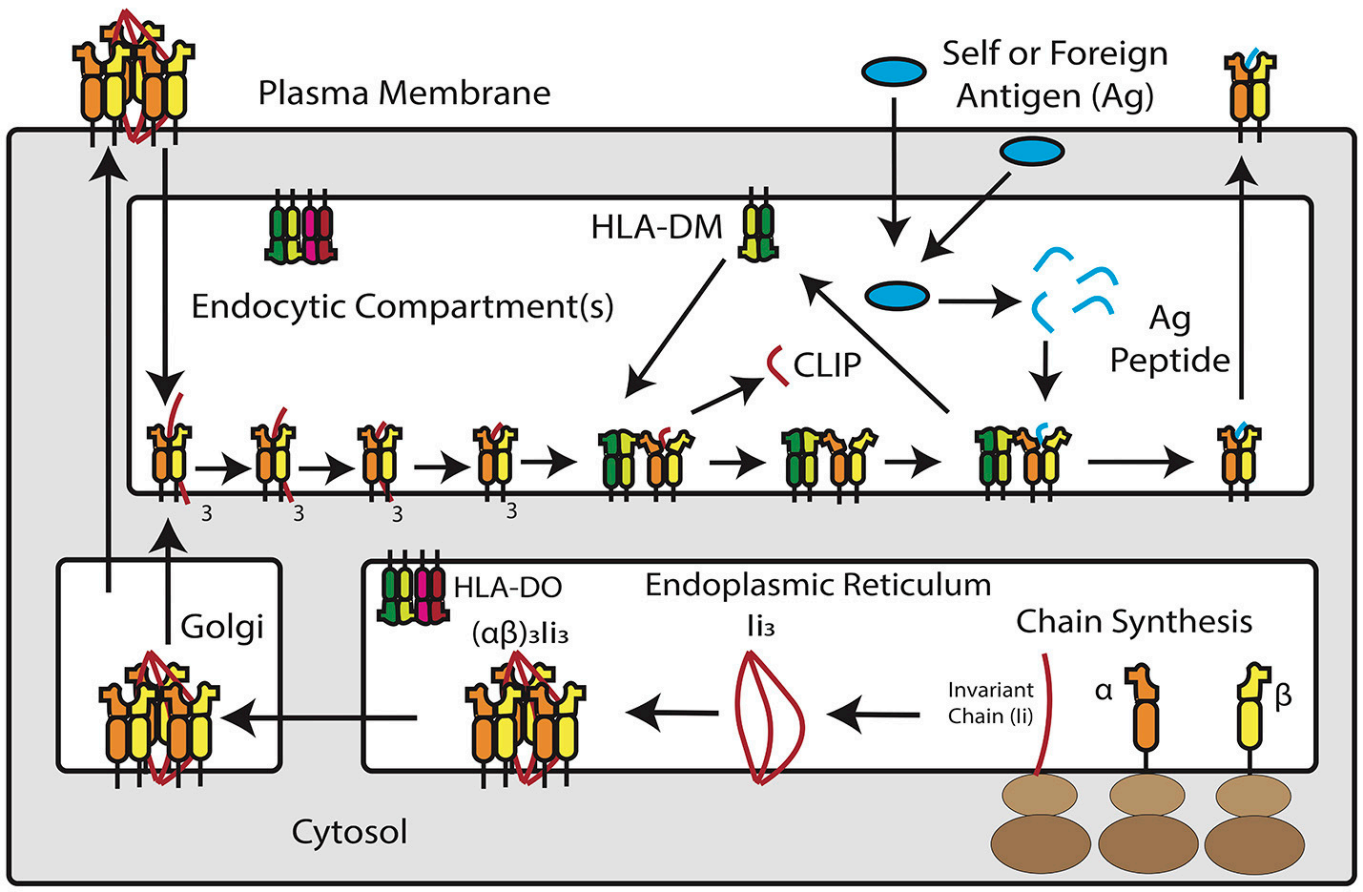
Overview of MHC Class II Antigen Presentation Pathway. Newly synthesized MHC class II α and β chains assemble into heterodimers in the endoplasmic reticulum, where they are bound by trimers of invariant chain. MHC class II and invariant chain form nonamers–or, according to recent studies, pentamers and heptamers–that traffic into an acidic endosomal compartment. Within this compartment, invariant chain is degraded down to class II invariant chain-associated peptide (CLIP), which occupies the peptide binding groove of the MHC class II molecule. HLA-DM, a non-classical MHC protein, catalyzes the removal of CLIP in exchange for high-affinity peptide binders derived from extracellular or cytosolic antigens. In a subset of antigen presenting cells, HLA-DM is blocked by HLA-DO, which competitively binds to HLA-DM and prevents it from interacting with MHC class II. Once loaded with peptide, MHC class II molecules traffic to the plasma membrane for inspection by CD4^+^ T cells.

The intestinal mucosa is composed of simple columnar epithelium that comprises a surface area of 200–300 m^2^ (1). This immense structure facilitates the absorption of nutrients in the small intestine and water in the large intestine, while also acting as a barrier and modulator of immunity (5). Intestinal epithelial cells (IECs) include multiple different specialized cells including enterocytes, goblet cells, Paneth cells, enteroendocrine cells, M cells and tuft cells, all of which have discrete functions (Figure 1). Enterocytes are the most abundant cell type in the gut epithelium and function to transcytose antimicrobial proteins and IgA as well as absorb nutrients (6). Goblet cells secrete mucus, resistin-like molecule β, which modifies T cell-mediated immunity, and trefoil factor, which promotes epithelial healing after injury; these cells have also been shown to participate in antigen delivery to dendritic cells (DCs) of the submucosa through specialized antigen passages (7, 8). Paneth cells in the small intestine secrete microbicidal proteins including α-defensins, C-type lectins, lysozyme and phospholipase A_2_; they also sustain stem cells in the crypts of Lieberkühn to promote epithelial regeneration (6). Enteroendocrine cells secrete neurohormones including gastric inhibitory peptide, glucagon-like peptide, and vasoactive intestinal peptide in response to nutrients in order to regulate motility and digestion (6). M cells (microfold cells), a specialized EC subset derived from enterocytes, transcytose antigens to the underlying gut-associated lymphoid tissue (GALT), the complement of lymphocytes that is composed of intraepithelial and lamina propria lymphocytes (IELs, LPLs) (9–11). Because of their role in antigen uptake, there has been interest in whether M cells present antigens using the MHC class II pathway to facilitate adaptive immunity, though existing evidence is conflicting (12–16). Tuft cells, a rare EC with a distinctive tufted morphology, express chemosensory receptors and may have roles in type 2 immunity and mucosal immunity, but remain poorly understood (17, 18).

IECs have a known role in innate immune responses via expression of a variety of pattern recognition receptors (PRRs). PRRs trigger intracellular pathways that lead to cytokine and chemokine release. PRRs important in the gut include Toll-like receptors 1–9 (TLRs) and nucleotide-binding oligomerization domain-containing proteins (NODs) that recognize pathogen-associated molecular patterns (PAMPs) derived from microbial components (19–21). Interestingly, apical TLRs on the luminal side of the gut (TLR 1 and 2) appear to be hyporesponsive to PAMPs *in vitro*, while the other PRRs expressed in IECs are either endosomal (TLR3–4, 7–9), cytoplasmic (NOD1 and NOD2) or only on the submucosal basolateral membrane (TLR4 and 5), supporting a role in tolerance to the gut microbiome (21–23).

The pseudostratified epithelium of the airway is ~100 m^2^ in surface area and composed of a variety of airway epithelial cells (AECs), many of which are specialized to the lung but homologous to the gut. The bronchial epithelium is composed of basal cells, columnar ciliated epithelial cells, mucous goblet cells, brush or tuft cells and Clara cells (24, 25). The alveolar epithelium, meanwhile, is composed mostly of Type I and Type II pneumocytes (24, 26). Neuroendocrine cells, similar to the gut, promote the vasomotor function of the airways (27). Basal cells, similar to Paneth cells, are important for epithelial regeneration and produce bioactive molecules including endopeptidase, 15-lipoxygenase products and cytokines (28, 29). Goblet cells, like those of the gut, secrete mucus in order to trap foreign particles and pathogens (24, 30). Columnar ciliated cells account for the majority of AECs in the bronchial lumen and are responsible mainly for mucus clearance (31). Clara cells produce surfactants and antiproteases including secretory leukocyte protease inhibitor and p450 mono-oxygenases (32). Type I pneumocytes are mainly responsible for gas exchange and make up the majority of the alveolar surface, though recent evidence suggests they may have additional roles in remodeling, regulation and defense (33). Type II pneumocytes are responsible for surfactant production and reuptake though they also act as progenitor cells and enhance immune responses (33, 34). Unlike the gut, an integrated mucosal immune system does not exist in the healthy adult human lung, though bronchus-associated lymphoid tissue (BALT) is present in young children and also develops in many disease states in adulthood (35, 36). AECs express a similar variety of PRRs as IECs including all known human TLRs, RIG-1-like receptors, NOD-like receptors, C-type lectins and surfactant proteins (26, 37–41).

### Overview of MHC Class II and costimulatory molecules

MHC class II molecules are transmembrane αβ heterodimers. In humans, there are three MHC class II isotypes: HLA-DR, HLA-DP, and HLA-DQ, encoded by α and β chain genes within the Human Leukocyte Antigen (HLA) locus on chromosome 6. The expression of MHC class II antigen presentation machinery is tightly regulated by class II transactivator (CIITA), which recruits DNA-binding factors, chromatin modifying proteins, and transcription initiators to the MHC II locus. The class II pathway for processing and presenting antigen is complex but involves interaction with accessory molecules and trafficking through intracellular compartments (42–44). In the ER, nascent MHC class II molecules associate with invariant chain (CD74), a dedicated chaperone protein that directs MHC class II into a low-pH, late-stage endosomal compartment, known as the MHC II compartment (MIIC). Within MIIC, proteases cleave invariant chain and leave a nested set of invariant chain fragments known as class II invariant chain-associated peptides (CLIP) (44). CLIP temporarily occupies the peptide binding groove of the MHC class II molecule. A catalytic protein, HLA-DM, exchanges CLIP for peptides that bind MHC class II with high-affinity (45). Some APCs, including B cells, thymic epithelial cells and certain DCs, express a regulator of HLA-DM, known as HLA-DO, which competitively inhibits DM-MHC class II interaction (46). Once formed, peptide/MHC class II complexes traffic to the cell surface for interaction with CD4^+^ T cells through the T-cell receptor, as the first signal to the lymphocyte required to elicit an antigen-specific adaptive response. The MHC class II pathway is shown schematically in Figure 1.

Efficient activation of naïve CD4^+^ T cells requires a second signal to the lymphocyte in the form of co-stimulation to complete the APC-T cell interaction. Classical costimulatory signals include CD80 and CD86, members of the B7 family that interact with stimulatory CD28 or inhibitory CTLA-4 on T cells (47). These molecules are upregulated on professional APCs in response to PAMPs or damage associated molecular patterns (DAMPs), such as ATP (48). T cell recognition of peptide/MHC without sufficient co-stimulation induces a hyporesponsive, anergic state (49).

## Potential role of ECs in antigen presentation

### MHC class II in health and disease in humans

#### Intestine

**Figure 2:**
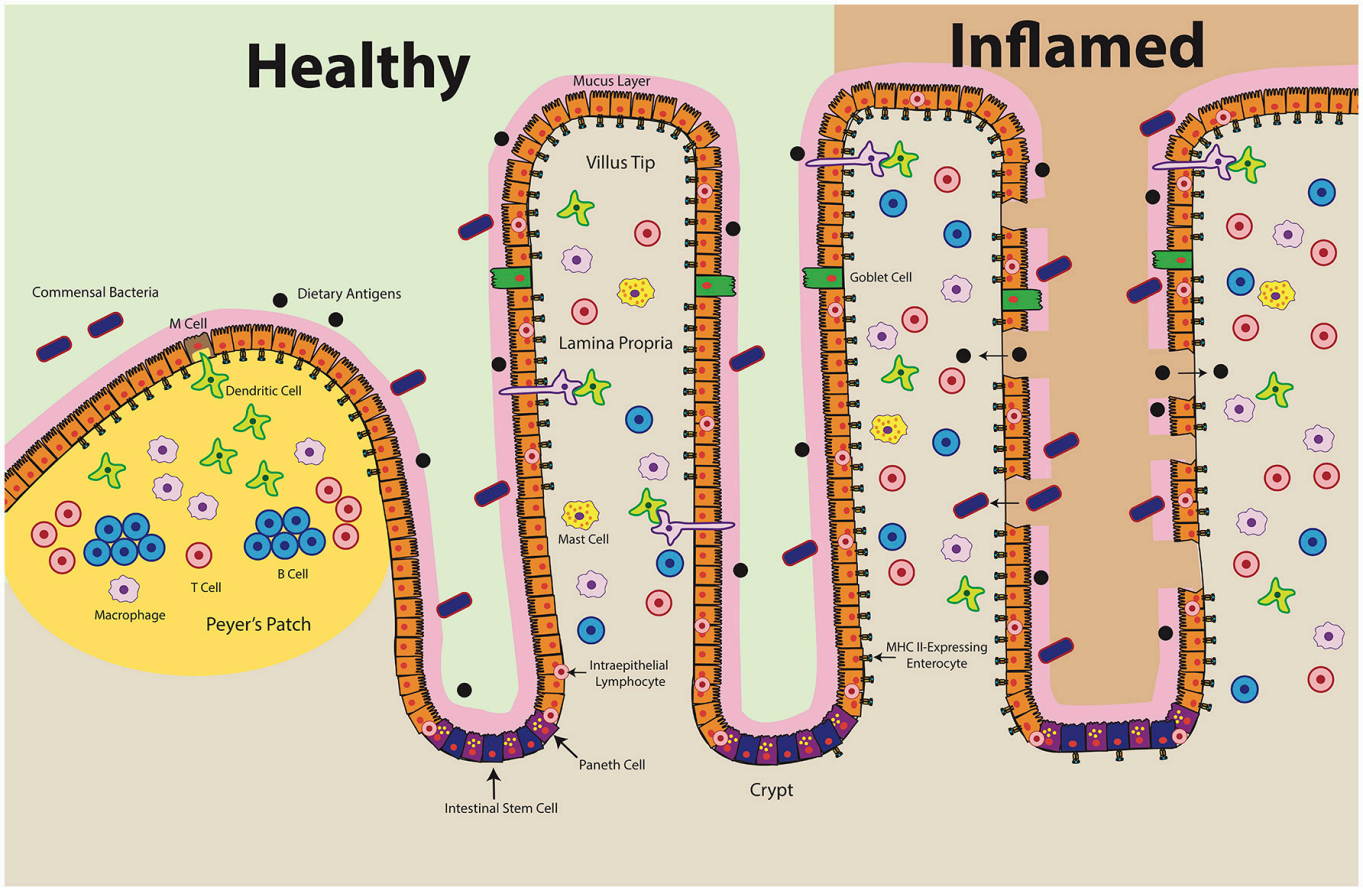
Intestinal Epithelial Cell MHC Class II Expression in Health and Disease. At homeostasis (left), MHC class II is constitutively expressed in the upper villi of the small intestine. At the crypt base, intestinal stem cells self-renew and differentiate into specialized cell types: antimicrobial-producing Paneth cells, mucus-producing Goblet cells, hormone-producing enteroendocrine cells, and nutrient-absorptive enterocytes (200). Healthy crypts lack MHC class II expression. Intraepithelial lymphocytes, consisting of T cells and γδ T cells, likely play a key role in maintaining the baseline expression of MHC class II in ECs by producing IFNγ. During disease (right), MHC class II levels increase and extend into the crypts. Epithelial barrier integrity decreases, which may result in ECs encountering antigen along both the apical and basolateral surfaces. Organized lymphoid structures, known as Peyer's Patches, contain dense concentrations of professional antigen presenting cells (B cells, macrophages, dendritic cells). These cells encounter antigen delivered by microfold (M) cells, which transcytose luminal antigens. Whereas MHC class II expression has been shown in the Peyer's Patch epithelium, there are conflicting reports regarding MHC class II expression by M cells.

IECs have been described as capable of MHC class II expression for several decades (50–54). MHC class II, HLA-DM and invariant chain have been reproducibly detected in IECs throughout all segments of the small intestine (12, 52, 54–57). In humans, cell surface expression of class II is first detected around 18 weeks' gestation and increases through development (15, 58, 59). At homeostasis, MHC class II appears to be constitutively expressed on small intestinal enterocytes, most densely in the upper villus (15, 53, 56). Conversely, MHC class II is absent from small intestinal crypts as well as colonic epithelium under normal physiologic conditions but is upregulated in specimens obtained from patients with active inflammatory bowel disease (IBD), celiac disease, and graft vs. host disease (Figure 2) (55, 60–66). Exposure to inflammatory antigens, such as gliadin in celiac disease, has also been shown to cause the upregulation of cell surface MHC class II (62, 67). These changes are dependent on active disease; celiac patients in remission have IEC MHC class II levels comparable to those of non-celiac controls (68). IFNγ appears to be the key disease-elevated cytokine that regulates this process (69). In IBD, for example, increased surface MHC class II expression is correlated with increased tissue IFNγ levels (Figure 2) (70).

IECs are polarized, with brush border enzymes localized to the apical (luminal) surface to break down dietary antigens and poly-Ig receptors restricted to the basolateral surface to translocate IgA into the intestinal lumen (71). This polarity is important as peptide-presentation to the resident immune cells of the GALT is necessary for systemic crosstalk. Some early tissue staining studies in humans showed predominantly apical expression of MHC class II in IECs (53, 55, 72). However, other reports, including a comparatively recent study, show lateral and basolateral MHC class II (73–75). These contradictory observations may be due to variability in methods of tissue processing and labeling, which has a significant effect on antigen stability and labeling efficiency (68, 76, 77). Notably, *in vitro* studies show expression of MHC class II along the basolateral surface and *in vivo* studies suggest that the amount of MHC class II along the basolateral surface of IECs is physiologically relevant (78–80).

Intestinal inflammation may also change MHC class II localization in IECs. Both conventional and electron microscopy have been used to show redistribution of IEC MHC class II from multivesicular bodies (late endosomes) to the basolateral membrane located on the submucosal side of the epithelial membrane in both celiac disease and IBD (74, 81). Increased trafficking of MHC class II to the cell surface likely requires downregulation of MARCH8 ubiquitin ligase, which drives MHC class II internalization and which IECs express at high levels (82). A similar pathway has been observed in DCs, where MARCH 1 is downregulated upon maturation stimulated by TLR ligands (83). Redistribution of MHC class II may allow IECs to influence immune responses during a pathogenic or inflammatory insult, by presenting peptides that promote immune clearance or induce tolerance.

Co-stimulatory molecules CD80 and CD86 are not expressed on IECs at baseline (57, 84, 85). Whether these molecules are expressed during inflammation is less clear. Some studies report that human IECs express neither CD80 nor CD86 during IBD, while others show selective expression of CD86 during active disease in biopsy specimens or with IFNγ-treatment in culture (85, 86). There is also evidence that the costimulatory molecule CD40, which interacts with CD40 ligand (CD40L) on T cells, is expressed by IECs during IBD in regions with visible pathology (87, 88). IECs may provide other forms of co-stimulation, such as CD58 (LFA-3), which interacts with CD2 on the surface of T cells (89). IECs express basolateral CD58 constitutively on surgically resected colonic epithelium and *in vitro* treatment with anti-CD58 antibody inhibits stimulation of antigen-specific CD4^+^ T cell clones by antigen-pulsed IECs in a dose-dependent manner in humans (90).

#### Lung

**Figure 3:**
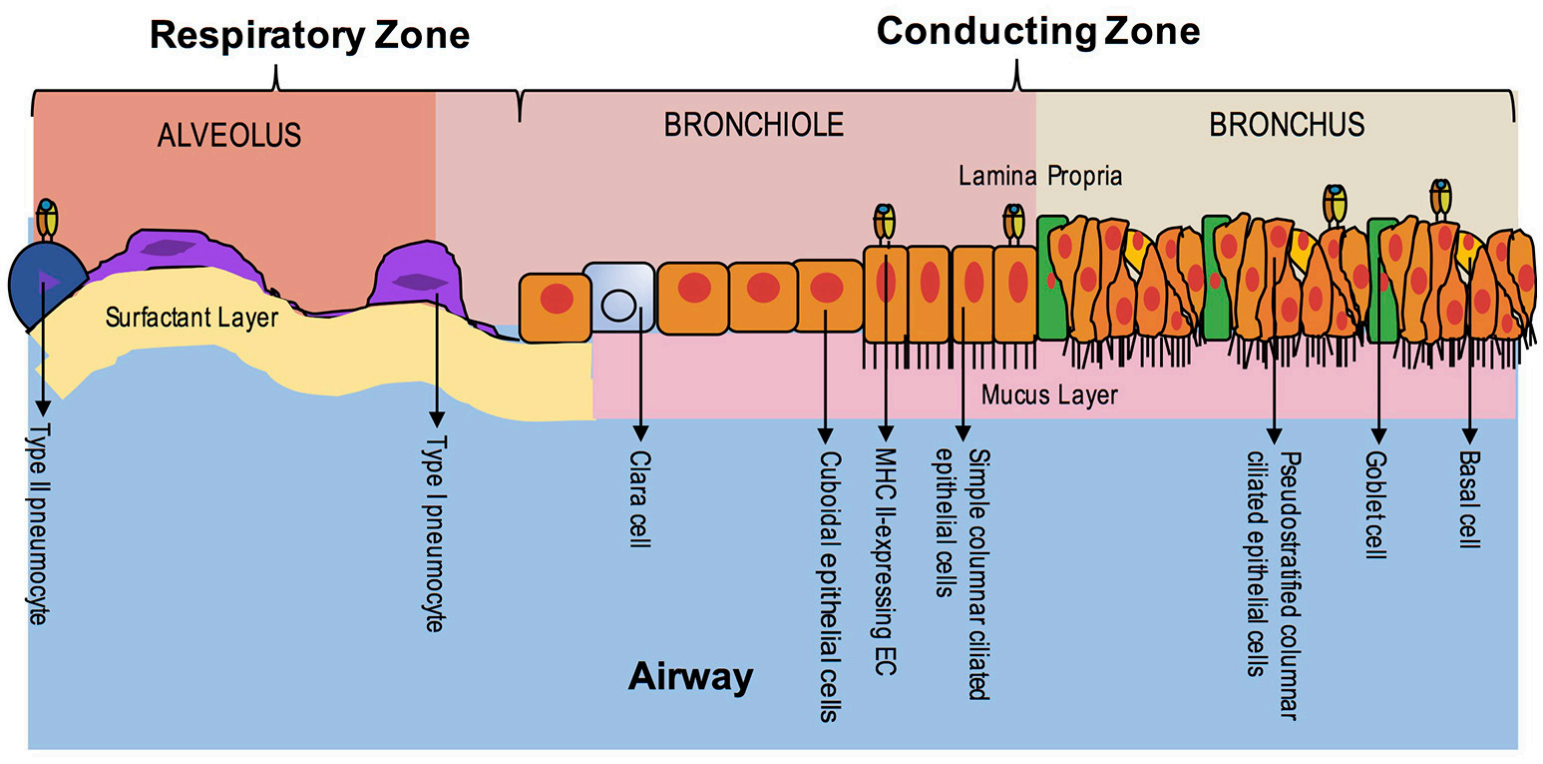
EC MHC Class II Expression in the Lung During Homeostasis. The airway is composed of the upper airway conducting zone for humidifying and clearing particulates of inhaled air (bronchi and bronchioles) and lower airway respiratory zone for gas exchange (respiratory bronchioles and alveoli). At homeostasis, MHC class II expression has been seen in the ciliated ECs of the upper airway and in Type II pneumocytes of the alveoli. The polarity of class II expression is not well-defined. Unlike the intestine, organized lymphoid structures are not found in adulthood, except in disease states.

Unlike the gut during ontogeny, fetal lung tissue does not appear to express MHC class II on AEC surfaces during gestation except in the case of active inflammation (91). Interestingly, invariant chain expression without co-expression of MHC class II has been detected on fetal alveolar epithelium by 12–14 weeks' gestational age in humans (92). Adult AECs, like small intestinal epithelium, were initially shown to constitutively express MHC class II on both bronchial and alveolar epithelium, specifically on type II pneumocytes and ciliated ECs (Figure 3) (93–95). However, additional studies utilizing clinical specimens have provided conflicting data, especially in primary bronchial EC cultures (96–99). Evidence in studies comparing germ-free to conventional rats supports constitutive surface expression of MHC II in lung parenchymal AECs, specifically Type II pneumocytes, but decreased expression in bronchial epithelium of germ-free rats, suggesting site-specific expression (100). Lung tissue obtained from patients with allergy or autoimmunity, including chronic bronchitis, asthma, idiopathic pulmonary fibrosis or lung transplant rejection, shows enhanced expression of MHC class II on AECs (96, 97, 101–103). Viral infection, including parainfluenza, have demonstrably increased AEC MHC class II expression, whereas bacterial infection appears to have the opposite effect in human lung specimens (91, 97, 104).

Co-stimulatory molecule expression appears to be region-specific in humans, as well. *in vitro* studies show baseline expression of CD86 on both bronchial and alveolar cells (A549 cell line), but baseline CD80 expression only on alveolar cells (98). Viral infection, specifically with rhinovirus, upregulates CD80 on alveolar cells and CD86 on bronchial cells (98). *In vivo* data obtained from lung biopsies in patients with a variety of autoimmune pathologies, including lung transplant rejection and idiopathic pulmonary fibrosis, shows increased expression of CD80 and CD86 on AEC from all segments of the respiratory tract (97, 105). In comparison, in bronchiolitis obliterans organizing pneumonia (now known as cryptogenic organizing pneumonia), an idiopathic interstitial lung disease believed to be secondary to epithelial damage, CD80 is upregulated in AECs without concurrent upregulation of CD86 or MHC class II expression (97, 106). Like gut, CD58 is constitutively expressed on alveolar ECs, though expression has not been demonstrated in isolated Type II pneumocytes (95).

### *In vitro* evidence for ECs as antigen presenting cells

Studies utilizing human IEC lines (T84 and HT29) show that IFNγ-treated, protein antigen-pulsed IECs can stimulate antigen-specific immune responses in T cell hybridomas (107). T cell hybridomas do not need co-stimulation, which arguably mimics the reduced costimulatory requirements of the majority of T cells in the lamina propria, which are antigen-experienced memory cells (108, 109). Follow-up work in IECs found that generation of specific MHC II-restricted peptide epitopes differed if antigen was taken up from the apical or basolateral IEC surface (78). During disease, inflammatory signals including IFNγ and TNFα in the gut increase epithelial permeability (Figure 2) (110–112). When the epithelium is breached, IECs may interact with antigen along both the apical and basolateral surfaces, raising the possibility that novel peptide epitopes can be generated. Dotan et al. found that IECs isolated from surgically resected colon of Crohn's disease and ulcerative colitis patients induced CD4^+^ T cells to proliferate and secrete more IFNγ than control IECs in a mixed lymphocyte reaction (65). This effect was blocked with an anti-HLA-DR antibody.

Another mechanism by which ECs may modulate antigen presentation is through exosomes. Exosomes, cell-derived vesicles laden with MHC class II, are released extracellularly when the limiting membrane of a multi-vesicular endosome fuses with the plasma membrane (113). In fact, several studies show that exosomes from IFNγ-treated IECs express elevated MHC class II (114–116). These exosomes express late-endosomal markers, consistent with their origin in multi-vesicular bodies. Evidence suggests that IEC exosomes do not directly stimulate antigen-specific T cells but are first acquired by DCs. DCs primed with IEC exosomes require lower doses of antigen to stimulate T cell hybridomas (116). Exosomal transfer of peptide/MHC II complexes may promote rapid, primary adaptive immune responses by equipping DCs to stimulate naive T cells. Defining the relative contributions of direct IEC antigen presentation vs. exosome release to intestinal tolerance and immunity will require further investigation and may provide important insights into the communication between gut and lung.

AECs isolated from bronchial epithelium in humans and cultured with IFNγ have been shown to trigger proliferation of allogeneic lymphocytes as well (99). Additionally, investigations by Cunningham et al. demonstrated that the addition of anti-CD28 antibody as a co-stimulatory signal allows allogeneic CD4^+^ T cells to proliferate in response to IFNγ-stimulated MHC class II^+^ AECs (117). Further characterization by other groups shows that purified allogeneic T cells are stimulated in response to bronchial ECs, which is abolished by the addition of anti-DR antibody (118). Bronchial ECs have also been shown to present protein antigens to antigen-specific sensitized T cells, suggesting the ability of AECs to process and present foreign antigen to the underlying lymphoid tissue (119). Experiments utilizing electron microscopy verify that AECs stimulated with IFNγ are able to endocytose antigen and that, like IECs, uptake is polarized on the luminal side of tissue explant cultures. Co-localization studies further demonstrate the trafficking of these antigens through early and late endosomes to acid vesicles and lysosomes (120).

*In vitro* studies have important caveats. MHC class II is only expressed in the large intestine and potentially bronchi during disease, yet many commonly used intestinal cell lines (Caco-2, T84, and HT29) are colon-derived and pulmonary cell lines (BEAS-2B) bronchial in origin. Therefore, studies using these cell lines may be more representative of EC antigen presentation during inflammation rather than homeostasis. Colorectal cancer cells are also susceptible to genetic and epigenetic abnormalities, including changes in DNA methylation that affect CIITA expression (121). Small intestinal EC lines, such as HEC-6 and H4, exist, but are derived from fetal tissue and are more representative of crypt stem cells than fully differentiated ECs (122). Additionally, AECs are often derived from bronchoalveolar lavage brushings or fluid in patients with additional underlying pathologies, which are highly operator- and patient-dependent and may not be representative of the entire airway epithelium. Furthermore, *in vitro* experiments using peripheral blood T cells may not recapitulate interactions between ECs and organ-specific T cells. For instance, one study shows that IECs induce CD4^+^ IELs to secrete IFNγ, but not CD4^+^ T cells from the lamina propria or spleen (123). Moreover, CD4^+^ and CD8^+^ T cells found in adult human mucosa, including in both gut and lung, are largely memory cells, requiring different stimuli than naïve cells (124). Therefore, the complexity of the epithelium and the arrangements of the many cell types found within may not be well-represented in cultures of primary purified cell lines.

### *In vivo* evidence for ECs as antigen presenting cells

Several *in vivo* studies of IEC antigen presentation have focused on IBD, where inflammatory responses to the gut microbiota are believed to elicit tissue damage, yet the role of IECs themselves remain poorly defined. Maggio-Price et al. induced colonic inflammation in RAG2^−/−^ mice exclusively expressing MHC class II either on IECs or DCs. Animals with MHC class II^+^ DCs developed severe colitis, whereas mice with MHC class II on IECs developed only mild inflammation (125). Additionally, mice lacking MHC class II on DCs appeared to develop intestinal inflammation due to lack of proper CD4^+^ T cell-mediated adaptive immune responses to commensal bacteria, as gnotobiotic mice under the same conditions did not develop inflammation (126). In a different murine colitis model, Thelemann et al. showed that selectively knocking out MHC class II in IECs worsened colitis; additionally, mice without IEC MHC class II had higher IFNγ levels and a reduced proportion of Tregs (80). In this system, IECs failed to express CD40, CD80, or CD86 co-stimulatory molecules. Another elegant study targeted hemagglutinin (HA) expression to IECs in transgenic mice expressing an HA-specific T cell receptor. This resulted in the expansion of HA-specific Tregs and was not dependent on DCs acquiring antigen from apoptotic IECs. Isolated primary IECs directly stimulated Treg proliferation in an MHC class II-dependent manner (79). Interestingly, the authors ruled out TGF-β and retinoic acid as effectors—molecules known to skew naive T cells into induced Tregs (127). Together, this suggests a tolerogenic role for IECs that is not dependent on co-stimulation of CD80 or CD86. Similar *in vivo* data has not been collected in the respiratory tract of animal models, and effects on the lung epithelium were not evaluated in the above models.

### Cytokine regulation

Potential immune cell sources of IFNγ that upregulate MHC class II include natural killer (NK) and natural killer T (NKT) cells, group 1 ILCs (ILC1s), γδ T cells, CD8^+^ T cells and subsets of CD4^+^ T cells, which comprise the makeup of the GALT (128–132). The pIV isoform of the MHC class II transactivator CIITA is the main form expressed in non-hematopoietic cells in response to interferon gamma (IFNγ) and has been found in IECs (133, 134). Adoptive transfer of CD4^+^ T cells into mice induces IEC MHC class II expression, whereas the transfer of IFNγ-knockout T cells does not (80). Direct treatment with IFNγ has been shown to increase AEC MHC II expression in rats both *in vitro* and *in vivo* (100, 119). These findings have been re-capitulated in human AECs *in vivo*, as well (99, 135). AECs may enhance MHC class II expression via a CIITA-independent pathway, at least when exposed to viral particles, though data appear conflicting (104, 136). A possible explanation for tissue-specific differences in cytokine regulation may be that IELs or other cell types that produce IFNγ to drive MHC class II in IECs, such as NK cells or ILC1, may be more abundant in the GALT compared to the airway, though further characterization is required (137–139).

To date, there is limited evidence that cytokines besides IFNγ induce MHC class II on non-hematopoietic cells during inflammation. One candidate is IL-27, an IL-12 superfamily cytokine released by activated DCs and elevated during intestinal inflammation (140). IL-27 elevates CIITA levels in colorectal cancer cells and stimulates endothelial cells *in vitro* to express HLA-DR, -DP, -DQ, -DM, and invariant chain (141, 142). The overlapping effects of IL-27 and IFNγ are consistent with the fact that both cytokines promote T helper 1 (Th1) CD4^+^ T cell responses (143).

Another potential candidate is IL-18. IL-18, originally known as IFNγ inducing factor or IGIF, is a member of the IL-1 cytokine family and can mediate both Th1- and Th2-type responses (144–147). IL-18 has been shown to be elevated in autoimmune colitis including IBD and celiac disease and is a key mediator of intestinal homeostasis (144, 148–152). IEC appear to constitutively produce IL-18, at least *in vitro*, and promote increased production of IFNγ by T cells obtained from patients with active inflammation (153, 154). Interestingly, tissue explants from patients with active celiac disease show IL-18 expression only in the crypts (151). In a murine model of an NLRC4 inflammasome mutation, the intestinal epithelium appears solely responsible for the systemic elevation of IL-18 seen in macrophage activation syndrome and, furthermore, is associated with upregulation of IFNγ-induced genes and multiple genes associated with antigen presentation in the intestine (155). Recent evidence in humans shows that AEC also constitutively produce IL-18 *in vitro* in animal models (156, 157). IL-18 has been shown to directly upregulate MHC class II expression on IFNγ-stimulated keratinocytes, but this has not yet been explored in intestinal or airway epithelium (158). Whether EC-produced IL-18 is involved in paracrine MHC class II upregulation along the crypt-to-villus axis or through directing GALT-mediated IFNγ-production also remains unknown. Therefore, further investigation is needed to determine if these or other region-specific cytokines upregulate EC MHC class II expression.

### Role of the microbiome

Commensal bacteria reside within the lumen of the gut, reaching a density of up to 10^12^ cells per cm^3^ in the large intestine (159). It is well-established that these microbes contribute to the development of the intestinal immune system; gnotobiotic mice, for example, do not form isolated lymphoid structures in the small intestine (160). Though the lung and gut share a common origin at the oropharynx, microbial populations are vastly different. The lung is not completely sterile but has a much lower bacterial burden without a characteristic microbiome like the gut; rather, lung flora tends to resemble oral flora and may change in response to a variety of stimuli and pathologies (161, 162).

Significantly, IECs of gnotobiotic mice lack MHC class II expression, while exposure to bacteria is found to increase IFNγ expression by γδ T cells and induce CIITA and MHC class II (163–165). There is limited but interesting evidence that specific classes of commensals, such as segmented filamentous bacteria, are sufficient to induce MHC class II in IECs (165). However, because γδ T cells compose a higher proportion of IELs in mice (~50%) than in humans (~10%), other cellular sources of class II inducing signals may be important in humans (166, 167). Additionally, the roles of viruses and fungi within the microbiome and their effects on EC MHC class II expression remain largely unexplored.

Recent evidence argues for a reciprocal effect of MHC class II in shaping the microbiome. Studies in natural fish populations link MHC class II allelic variation with the abundance of certain microbial taxa (168). These findings corroborate studies in laboratory mice, which show that MHC class II-linked changes in the microbiome mediate risk of enteric infection and autoimmune disease, such as type 1 diabetesc (169, 170). The precise mechanisms behind these effects remain poorly understood, though there is evidence that MHC class II polymorphisms control microbial populations through IgA phenotype and thus modify susceptibility to pathogens (169).

Exploration into the “gut-lung axis” in which the microbiome of the gut has direct impact on susceptibility to pulmonary disease is of key interest (171). The gut microbiome has been shown to affect lung susceptibility to infection with viral, fungal and bacterial pathogens (172–176). The severity of ozone-induced asthma in mice appears to be regulated by the gut microbiome through short chain fatty acid production (177). Segmented filamentous bacteria, a gut commensal that induces MHC class II on IECs as above, have independently been shown to provoke autoimmunity of the lung epithelium, but whether this is through affecting MHC class II expression on AECs is unknown (165, 176). The microbiome may even affect predisposition to lung cancer as evidenced through murine studies focused on probiotic use, though further mechanistic and human studies are still needed in this area, as well (171, 178).

## Discussion

Though much of the available evidence on MHC class II expression by ECs was obtained decades ago, this is an exciting time for research into the role of ECs in mucosal immunology. Renewed awareness of the role played by epithelial cells in homeostasis and disease and technical advances in different areas open up several new avenues for research and clinical applications.

Celiac disease, in which blunting of the villous tips on biopsy is pathognomonic, provides an example of a disease in which the role of the EC should be re-visited. Pathogenic CD4^+^ T cells in celiac disease are DQ2- or DQ8-restricted, and T cell bound to DQ/gliadin tetramers are detectable using flow cytometry (179–182). Yet, most MHC class II studies examined in this review focus on HLA-DR. Cell surface HLA-DP and -DQ levels have been reported as lower, but T cells are remarkably sensitive, requiring as few as one to ten peptides per MHC II complexes for activation (183). Levels of MHC class II that are below the limit of detection by immunohistochemistry (used in many early papers) may therefore be sufficient to activate T cells. More sensitive techniques, such as flow cytometry or electron microscopy, are more informative, as evidenced by more recent papers. Another novel possibility is investigation utilizing multiplexed ion beam imaging (MIBI) to visualize large panels of cell-surface proteins tagged with elemental metals that may allow improved detection of MHC II isoforms and co-localization of various co-stimulatory molecules on tissue sections (184). Using these technologies to study celiac disease, a model disease in which the inciting immunogen and the presenting MHC class II-molecules are known, may provide important insights into the role of ECs in antigen presentation.

The function of co-stimulatory molecules in this process is another area that requires more investigation. While some description of EC surface expression of classic B7 molecules, CD80 and CD86, is found in the literature (above), their roles during homeostasis and inflammation remain unclear. The lack of expression of CD86 found in the gut, compared to constitutive expression in the airway, may suggest a diminished role of IECs in interactions with Tregs (185). Another avenue of exploration are other members of the B7 family that appear to have novel roles on non-professional antigen presenting cells, including ICOS ligand, PD-L1 (B7-H1), PD-L2 (B7-DC), B7-H3, and B7-H4. Work on the ICOS co-stimulation pathway in the airway already has provided promising results, with anti-ICOS treatment leading to prevention of chronic lung transplant rejection and obliterative bronchiolitis as well as ICOS being shown as an important player in asthma (186, 187). However, the contribution of the aerodigestive epithelium in mediating these interactions remains to be explored.

Further delineation of the subsets and character of ECs are needed as well. The epithelium is composed of both stem cells and specialized subtypes as described above, many of which remain poorly understood. Both MHC II expression and antigen presenting capabilities and function may therefore differ among these cells. Work reviewed here has shown that, for example, M cells in the gut or type II pneumocytes in the lung may have roles in antigen presentation and expression of HLA-DR (9, 11, 16). Furthermore, the polarity and anatomic localization of intestinal and pulmonary ECs also likely bear significant implications for antigen uptake, processing and presentation and warrant further investigation (53, 55, 71–73, 78–80). Defining the roles of these various cell types and their locoregional interactions thus may provide additional important insights.

The conditions under which mucosal MHC class II contributes to inflammation vs. tolerance also remain to be clearly delineated. The work by Westendorf et al. in mouse models shows direct expansion of Tregs in response to ECs, arguing that mucosal MHC class II can function in a tolerogenic role, though the work by Dotan suggests that increased MHC class II on IECs from IBD patients may more efficiently activate effector T cell responses leading to inflammation (65, 79, 188). Moreover, it is plausible that MHC class II on ECs not only allows ECs to modulate immune responses, but also in fact allows the immune system to regulate the epithelium. Cytokines released by adjacent mucosal and intraepithelial immune cells in response to EC presentation of MHC class II-bound peptides may alter cell renewal, barrier integrity, cell type composition, and the innate immune functions of the epithelium. A promising approach to explore these questions is in organoids derived from stem cells or induced pluripotent stem cells that can differentiate into specialized cell types mimicking the physiological structure of the epithelium (189–192). Organoids offer a reductionist setting for testing the role of immune cells, cytokines, pathogens, and other regulatory factors on MHC class II in primary ECs in a way that appears to model organs physiologically. For example, mouse intestinal organoids treated with IFN have been shown to upregulate MHC class II (193). Intestinal organoids can be readily infected with human strains of enteric pathogens, such as rotavirus, norovirus, and *Salmonella* to allow exploration of MHC class II internalization and polarity during infection and inflammation (194–196). The study of lung organoids remains, comparatively, in its infancy, though work has been done to create structures resembling fetal lung buds in the second trimester of gestation for the study of respiratory syncytial virus (197). Organoids may provide a model system to study the aforementioned hypotheses to provide evidence more pertinent to humans.

Finally, current research is actively exploring the contributions of the microbiome to systemic immunity. However, how the microbiome changes EC structure and function, especially through MHC class II and co-stimulatory molecule expression, and whether this affects development of disease and ultimate outcomes are also key questions. Highlighting the importance of co-stimulatory molecules, recent clinical work has demonstrated that the efficacy of cancer immunotherapies targeting B7 molecules PD-L1 or CTLA-4 in epithelial cancers including non-small cell lung carcinoma as well as colon cancer appear keenly dependent on the gut microbiome; lack of or depletion of commensals using oral antibiotics appears to attenuate tumor response to these therapies (198, 199). Interestingly, blockade of CTLA-4 on IELs led to IEC apoptosis in intestinal organoids, also suggesting a bidirectional trophic communication between ECs and effector immune cells through co-stimulatory molecules (199). Valuable lessons may be learned by comparing MHC class II expression on ECs in the intestinal and respiratory tracts.

Ultimately, systems that integrate immunological, microbial, and environmental signals to study EC MHC class II expression and function are likely to advance our understanding of mucosal immunity and the epithelium of the aerodigestive tract. How these findings can be manipulated to affect infectious, autoimmune or even neoplastic diseases will likely be pursued in the coming years.

## Author contributions

JW, DM, and EM were involved in planning the project. JW, DM, CM, and EM contributed to the writing of the final manuscript.

### Conflict of interest statement

The authors declare that the research was conducted in the absence of any commercial or financial relationships that could be construed as a potential conflict of interest.

## References

[1] de Santa Barbara P van den Brink GR Roberts DJ. Molecular etiology of gut malformations and diseases. Am J Med Genet. (2002) 115:221–30. 10.1002/ajmg.10978 12503117

[2] Metzger R Wachowiak R Kluth D. Embryology of the early foregut. Semin Pediatr Surg. (2011) 20:136–44. 10.1053/j.sempedsurg.2011.03.004 21708333

[3] Gilbert S Developmental Biology, 6th edn Sunderland: Sinauer Associates (2000).

[4] Mulholland M Lillemoe KD Doherty GM Maier RV Simeone DM Upchurch Jr GR. Greenfield's Surgery: Scientific Principles and Practice, 5th edn Vol. 1 Philadelphia, PA: Wolters Kluwer Health (2011).

[5] MacDonald TT Monteleone G. Immunity, inflammation & allergy in the gut. Science (2005) 307:1920–5. 10.1126/science.1106442 15790845

[6] Hooper LV. Epithelial cell contributions to intestinal immunity. Adv Immunol. (2015) 126:129–72. 10.1016/bs.ai.2014.11.003 25727289

[7] McDole JR Wheeler LW McDonald KG Wang B Konjufca V Knoop KA. Goblet cells deliver luminal antigen to CD103^+^ dendritic cells in the small intestine. Nature (2012) 483:345–9. 10.1038/nature10863 22422267 PMC3313460

[8] Knoop KA McDonald KG McCrate S McDole JR Newberry RD. Microbial sensing by goblet cells controls immune surveillance of luminal antigens in the colon. Mucosal Immunol. (2015) 8:198–210. 10.1038/mi.2014.58 25005358 PMC4268115

[9] Schulz O Pabst O. Antigen sampling in the small intestine. Trends Immunol. (2013) 34:155–61. 10.1016/j.it.2012.09.006 23083727

[10] Niess JH Brand S Gu X Landsman L Jung S McCormick BA. CX3CR1-mediated dendritic cell access to the intestinal lumen and bacterial clearance. Science (2005) 307:254–8. 10.1126/science.1102901 15653504

[11] Kerneis S Bogdanova A Kraehenbuhl JP Pringault E. Conversion by Peyer's patch lymphocytes of human enterocytes into M cells that transport bacteria. Science (1997) 277:949–52. 9252325 10.1126/science.277.5328.949

[12] Mayrhofer G Pugh CW Barclay AN. The distribution, ontogeny and origin in the rat of Ia-positive cells with dendritic morphology and of Ia antigen in epithelia, with special reference to the intestine. Eur J Immunol. (1983) 13:112–22. 10.1002/eji.1830130206 6403355

[13] Spencer J Finn T Isaacson PG. Expression of HLA-DR antigens on epithelium associated with lymphoid tissue in the human gastrointestinal tract. Gut (1986) 27:153–7. 10.1136/gut.27.2.153 3456338 PMC1433205

[14] Bjerke K Brandtzaeg P. Lack of relation between expression of HLA-DR and secretory component (SC) in follicle-associated epithelium of human Peyer's patches. Clin Exp Immunol. (1988) 71:502–7. 3289802 PMC1541660

[15] MacDonald T Weinel A Spencer J. HLA-DR expression in human fetal intestinal epithelium. Gut (1988) 29:1342–8. 3143626 10.1136/gut.29.10.1342 PMC1433992

[16] Nagura H Ohtani H Masuda T Kimura M Nakamura S. HLA-DR expression on M cells overlying Peyer's patches is a common feature of human small intestine. Acta Pathol Jpn. (1991) 41:818–23. 1785342 10.1111/j.1440-1827.1991.tb01624.x

[17] Gerbe F Sidot E Smyth DJ Ohmoto M Matsumoto I Dardalhon V. Intestinal epithelial tuft cells initiate type 2 mucosal immunity to helminth parasites. Nature (2016) 529:226–30. 10.1038/nature16527 26762460 PMC7614903

[18] Banerjee A McKinley ET von Moltke J Coffey RJ Lau KS. Interpreting heterogeneity in intestinal tuft cell structure and function. J Clin Invest. (2018) 128:1711–9. 10.1172/JCI120330 29714721 PMC5919882

[19] Okumura R Takeda K. Roles of intestinal epithelial cells in the maintenance of gut homeostasis. Exp Mol Med. (2017) 49:e338. 10.1038/emm.2017.20 28546564 PMC5454438

[20] Okumura R Takeda K. Maintenance of intestinal homeostasis by mucosal barriers. Inflamm Regen. (2018) 38:5. 10.1186/s41232-018-0063-z 29619131 PMC5879757

[21] Fukata M Arditi M. The role of pattern recognition receptors in intestinal inflammation. Mucosal Immunol. (2013) 6:451–63. 10.1038/mi.2013.13 23515136 PMC3730813

[22] Otte JM Cario E Podolsky DK. Mechanisms of cross hyporesponsiveness to Toll-like receptor bacterial ligands in intestinal epithelial cells. Gastroenterology (2004) 126:1054–70. 10.1053/j.gastro.2004.01.007 15057745

[23] Melmed G Thomas LS Lee N Tesfay SY Lukasek K Michelsen KS. Human intestinal epithelial cells are broadly unresponsive to Toll-like receptor 2-dependent bacterial ligands: implications for host-microbial interactions in the gut. J Immunol. (2003) 170:1406–15. 10.4049/jimmunol.170.3.1406 12538701

[24] Tam A Wadsworth S Dorscheid D Man SF Sin DD. The airway epithelium: more than just a structural barrier. Ther Adv Respir Dis. (2011) 5:255–73. 10.1177/1753465810396539 21372121

[25] Reid L Meyrick B Antony VB Chang LY Crapo JD Reynolds HY. The mysterious pulmonary brush cell: a cell in search of a function. Am J Respir Crit Care Med. (2005) 172:136–9. 10.1164/rccm.200502-203WS 15817800 PMC2718446

[26] Leiva-Juarez MM Kolls JK Evans SE. Lung epithelial cells: therapeutically inducible effectors of antimicrobial defense. Mucosal Immunol. (2018) 11:21–34. 10.1038/mi.2017.71 28812547 PMC5738267

[27] Adriaensen D Brouns I Pintelon I De Proost I Timmermans JP. Evidence for a role of neuroepithelial bodies as complex airway sensors: comparison with smooth muscle-associated airway receptors. J Appl Physiol. (1985) (2006) 101:960–970. 10.1152/japplphysiol.00267.2006 16741263

[28] Hong KU Reynolds SD Watkins S Fuchs E Stripp BR. *In vivo* differentiation potential of tracheal basal cells: evidence for multipotent and unipotent subpopulations. Am J Physiol Lung Cell Mol Physiol. (2004) 286:L643–9. 10.1152/ajplung.00155.2003 12871857

[29] Knight DA Holgate ST. The airway epithelium: structural and functional properties in health and disease. Respirology (2003) 8:432–446. 10.1046/j.1440-1843.2003.00493.x 14708552

[30] Jeffery PK. Morphologic features of airway surface epithelial cells and glands. Am Rev Respir Dis. (1983) 128:S14–20. 10.1164/arrd.1983.128.2P2.S14 6881701

[31] Spina D. Epithelium smooth muscle regulation and interactions. Am J Respir Crit Care Med. (1998) 158:S141–5. 10.1164/ajrccm.158.supplement_2.13tac100a 9817737

[32] De RW Willems L Van GM Franken C Fransen J Dijkman J. Ultrastructural localization of bronchial antileukoprotease in central and peripheral human airways by a gold-labeling technique using monoclonal antibodies. Am Rev Respir Dis. (1986) 133:882–90. 3706899

[33] Dobbs LG Johnson MD. Alveolar epithelial transport in the adult lung. Respir Physiol Neurobiol. (2007) 159:283–300. 10.1016/j.resp.2007.06.011 17689299

[34] Wright JR. Immunoregulatory functions of surfactant proteins. Nat Rev Immunol. (2005) 5:58–68. 10.1038/nri1528 15630429

[35] Hiller AS Tschernig T Kleemann WJ Pabst R. Bronchus-associated lymphoid tissue (BALT) and larynx-associated lymphoid tissue (LALT) are found at different frequencies in children, adolescents and adults. Scand J Immunol. (1998) 47:159–62. 9496692 10.1046/j.1365-3083.1998.00276.x

[36] Tschernig T Pabst R. Bronchus-associated lymphoid tissue (BALT) is not present in the normal adult lung but in different diseases. Pathobiology (2000) 68:1–8. 10.1159/000028109 10859525

[37] Gay NJ Symmons MF Gangloff M Bryant CE. Assembly and localization of Toll-like receptor signalling complexes. Nat Rev Immunol. (2014) 14:546–58. 10.1038/nri3713 25060580

[38] Evans SE Xu Y Tuvim MJ Dickey BF. Inducible innate resistance of lung epithelium to infection. Annu Rev Physiol. (2010) 72:413–35. 10.1146/annurev-physiol-021909-135909 20148683 PMC4471865

[39] Opitz B van Laak V Eitel J Suttorp N. Innate immune recognition in infectious and noninfectious diseases of the lung. Am J Respir Crit Care Med. (2010) 181:1294–309. 10.1164/rccm.200909-1427SO 20167850

[40] Heyl KA Klassert TE Heinrich A Müller MM Klaile E Dienemann H. Dectin-1 is expressed in human lung and mediates the proinflammatory immune response to nontypeable Haemophilus influenzae. MBio (2014) 5:e01492-01414. 10.1128/mBio.01492-14. 25161190 PMC4173778

[41] Nayak A Dodagatta-Marri E Tsolaki AG Kishore U. An insight into the diverse roles of surfactant proteins, SP-A and SP-D in innate and adaptive immunity. Front Immunol. (2012) 3:131. 10.3389/fimmu.2012.00131 22701116 PMC3369187

[42] Jones PP Murphy DB Hewgill D McDevitt HO. Detection of a common polypeptide chain in IA and IE sub-region immunoprecipitates. Mol Immunol. (1979) 16:51–60. 376435 10.1016/0161-5890(79)90027-0

[43] Cloutier M Gauthier C Fortin JS Geneve L Kim K Gruenheid S. ER egress of invariant chain isoform p35 requires direct binding to MHCII molecules and is inhibited by the NleA virulence factor of enterohaemorrhagic Escherichia coli. Hum Immunol. (2015) 76:292–6. 10.1016/j.humimm.2015.02.002 25731712

[44] Neefjes J Jongsma MLM Paul P Bakke O. Towards a systems understanding of MHC class I and MHC class II antigen presentation. Nat Rev Immunol. (2011) 11:823–36. 10.1038/nri3084 22076556

[45] Mellins ED Stern LJ. HLA-DM and HLA-DO, key regulators of MHC-II processing and presentation. Curr Opin Immunol. (2014) 26:115–22. 10.1016/j.coi.2013.11.005 24463216 PMC3944065

[46] Guce AI Mortimer SE Yoon T Painter CA Jiang W Mellins ED. HLA-DO acts as a substrate mimic to inhibit HLA-DM by a competitive mechanism. Nat Struct Mol Biol. (2013) 20:90–8. 10.1038/nsmb.2460 23222639 PMC3537886

[47] Greenwald RJ Freeman GJ Sharpe AH. The B7 family revisited. Annu Rev Immunol. (2005) 23:515–48. 10.1146/annurev.immunol.23.021704.115611 15771580

[48] Hemmi H Takeuchi O Kawai T Kaisho T Sato S Sanjo H. A toll-like receptor recognizes bacterial DNA. Nature (2000) 408:740–5. 10.1038/35047123 11130078

[49] Alegre M-L Frauwirth KA Thompson CB. T-cell regulation by CD28 and CTLA-4. Nat Rev Immunol. (2001) 1:220–8. 10.1038/35105024 11905831

[50] Ramig RF. Pathogenesis of intestinal and systemic rotavirus infection. J Virol. (2004) 78:10213–20. 10.1128/JVI.78.19.10213-10220.2004 15367586 PMC516399

[51] Peterson LW Artis D. Intestinal epithelial cells: regulators of barrier function and immune homeostasis. Nat Rev Immunol. (2014) 14:141–53. 10.1038/nri3608 24566914

[52] Wiman K Curman B Forsum U Klareskog L MalmnäS-Tjernlund U Rask L. Occurrence of Ia antigens on tissues of non-lymphoid origin. Nature (1978) 276:711–3. 366434 10.1038/276711a0

[53] Scott H Solheim BG Brandtzaeg P Thorsby E. HLA-DR-like antigens in the epithelium of the human small intestine. Scand J Immunol. (1980) 12:77–82. 10.1111/j.1365-3083.1980.tb00043.x 6997989

[54] Parr EL McKenzie IFC Demonstration of Ia antigens on mouse intestinal epithelial cells by immunoferritin labeling. Immunogenetics (1979) 8:499–508. 10.1007/bf01561459

[55] Chiba M Iizuka M Masamune O. Ubiquitous expression of HLA-DR antigens on human small intestinal epithelium. Gastroenterol Jpn. (1988) 23:109–16. 10.1007/bf02799021 3290039

[56] Lin XP Almqvist N Telemo E. Human small intestinal epithelial cells constitutively express the key elements for antigen processing and the production of exosomes. Blood Cells Mol Dis. (2005) 35:122–8. 10.1016/j.bcmd.2005.05.011 16027013

[57] Byrne B Madrigal-Estebas L McEvoy A Carton J Doherty DG Whelan A. Human duodenal epithelial cells constitutively express molecular components of antigen presentation but not costimulatory molecules. Hum Immunol. (2002) 63:977–86. 10.1016/S0198-8859(02)00436-6 12392850

[58] Oliver AM Thomson AW Sewell HF Abramovch DR. Major histocompatibility complex (MHC) class II antigen (HLA-DR, DQ & DP) expression in human fetal endocrine organs and gut. Scand J Immunol. (1988) 27:731–7. 10.1111/j.1365-3083.1988.tb02407.x 3293192

[59] Natali PG De Martino C Pellegrino MA Ferrone S. Analysis of the expression of I-Ak-like antigens in murine fetal and adult tissues with the monoclonal antibody 10–2.16. Scand J Immunol. (1981) 13:541–6. 10.1111/j.1365-3083.1981.tb00167.x 6171869

[60] Koretz K Momburg F Otto HF Moller P. Sequential induction of MHC antigens on autochthonous cells of ileum affected by Crohn's disease. Am J Pathol. (1987) 129:493–502. 3425689 PMC1899812

[61] Momburg F Koretz K Von Herbay A Moller P. Nonimmune human cells can express MHC class II antigens in the absence of invariant chain–an immunohistological study on normal and chronically inflamed small intestine. Clin Exp Immunol. (1988) 72:367–72. 3048805 PMC1541582

[62] Fais S Maiuri L Pallone F De Vincenzi M De Ritis G Troncone R. Gliadin induced changes in the expression of MHC-class II antigens by human small intestinal epithelium. Organ culture studies with coeliac disease mucosa. Gut (1992) 33:472–5. 1582589 10.1136/gut.33.4.472 PMC1374061

[63] Kelly J O'Farrelly C O'Mahony C Weir D Feighery C. Immunoperoxidase demonstration of the cellular composition of the normal and coeliac small bowel. Clin Exp Immunol. (1987) 68:177. 2820630 PMC1542674

[64] Mason DW Dallman M Barclay AN. Graft-versus-host disease induces expression of Ia antigen in rat epidermal cells and gut epithelium. Nature (1981) 293:150–1. 7022229 10.1038/293150a0

[65] Dotan I Allez M Nakazawa A Brimnes J Schulder-Katz M Mayer L. Intestinal epithelial cells from inflammatory bowel disease patients preferentially stimulate CD4^+^ T cells to proliferate and secrete interferon-γ. Am J Physiol. Gastrointest Liver Physiol. (2007) 292, G1630–40. 10.1152/ajpgi.00294.2006 17347451

[66] Mayer L Eisenhardt D Salomon P Bauer W Plous R Piccinini L. Expression of class II molecules on intestinal epithelial cells in humans. Differences between normal and inflammatory bowel disease. Gastroenterology (1991) 100:3–12. 1983847 10.1016/0016-5085(91)90575-6

[67] Zimmer KP Poremba C Weber P Ciclitira PJ Harms E. Translocation of gliadin into HLA-DR antigen containing lysosomes in coeliac disease enterocytes. Gut (1995) 36:703–9. 7797120 10.1136/gut.36.5.703 PMC1382673

[68] Arnaud-Battandier F Cerf-Bensussan N Amsellem R Schmitz J. Increased HLA-DR expression by enterocytes in children with celiac disease. Gastroenterology (1986) 91:1206–12. 10.1016/S0016-5085(86)80018-X 3758613

[69] Colgan SP Resnick MB Parkos CA Delp-Archer C McGuirk D Bacarra AE. IL-4 directly modulates function of a model human intestinal epithelium. J Immunol. (1994) 153:2122–9. 7914217

[70] Niessner M Volk BA. Altered Th1/Th2 cytokine profiles in the intestinal mucosa of patients with inflammatory bowel disease as assessed by quantitative reversed transcribed polymerase chain reaction (RT-PCR). Clin Exp Immunol. (1995) 101:428–35. 10.1111/j.1365-2249.1995.tb03130.x 7664489 PMC1553229

[71] Rojas R Apodaca G. Immunoglobulin transport across polarized epithelial cells. Nat Rev Mol Cell Biol. (2002) 3:944–56. 10.1038/nrm972 12461560

[72] Daar AS Fuggle SV Fabre JW Ting A Morris PJ. The detailed distribution of class II antigens in normal human organs. Transplantation (1984) 38:293–8. 6591602 10.1097/00007890-198409000-00019

[73] Hirata I Austin LL Blackwell WH Weber JR Dobbins WO III. Immunoelectron microscopic localization of HLA-DR antigen in control small intestine and colon and in inflammatory bowel disease. Digest Dis Sci. (1986) 31:1317–30. 3542442 10.1007/BF01299810

[74] Sarles J Gorvel JP Olive D Maroux S Mawas C Giraud F. Subcellular localization of class I (A,B,C) and class II (DR and DQ) MHC antigens in jejunal epithelium of children with coeliac disease. J Pediatr Gastroenterol Nutr. (1987) 6:51–6. 3540260 10.1097/00005176-198701000-00010

[75] Hundorfean G Zimmer KP Strobel S Gebert A Ludwig D Büning J. Luminal antigens access late endosomes of intestinal epithelial cells enriched in MHC I and MHC II molecules: *in vivo* study in Crohn's ileitis. Am J Physiol Gastrointest Liver Physiol. (2007) 293:G798–808. 10.1152/ajpgi.00135.2007 17673546

[76] Arnold MM Srivastava S Fredenburgh J Stockard CR Myers RB Grizzle WE. Effects of fixation and tissue processing on immunohistochemical demonstration of specific antigens. Biotech Histochem. (1996) 71:224–30. 8896794 10.3109/10520299609117164

[77] Shi SR Liu C Pootrakul L Tang L Young A Chen R. Evaluation of the value of frozen tissue section used as “gold standard” for immunohistochemistry. Am J Clin Pathol. (2008) 129:358–66. 10.1309/7cxuyxt23e5al8kq 18285257

[78] Hershberg RM Cho DH Youakim A Bradley MB Lee JS Framson PE. Highly polarized HLA class II antigen processing and presentation by human intestinal epithelial cells. J Clin Invest. (1998) 102:792–803. 9710448 10.1172/JCI3201 PMC508942

[79] Westendorf AM Fleissner D Groebe L Jung S Gruber AD Hansen W. CD4^+^Foxp3^+^ regulatory T cell expansion induced by antigen-driven interaction with intestinal epithelial cells independent of local dendritic cells. Gut (2009) 58:211–9. 10.1136/gut.2008.151720 18832523

[80] Thelemann C Eren RO Coutaz M Brasseit J Bouzourene H Rosa M. Interferon-γ induces expression of MHC class II on intestinal epithelial cells and protects mice from colitis. PLoS ONE (2014) 9:e86844. 10.1371/journal.pone.0086844 24489792 PMC3904943

[81] Bar F Sina C Hundorfean G Pagel R Lehnert H Fellermann K. Inflammatory bowel diseases influence major histocompatibility complex class I (MHC I) and II compartments in intestinal epithelial cells. Clin Exp Immunol. (2013) 172:280–9. 10.1111/cei.12047 23574324 PMC3628330

[82] Uhlen M Fagerberg L Hallstrom BM Lindskog C Oksvold P Mardinoglu A. Tissue-based map of the human proteome. Science (2015) 347:1260419. 10.1126/science.1260419 25613900

[83] Walseng E Furuta K Goldszmid RS Weih KA Sher A Roche PA. Dendritic cell activation prevents MHC class II ubiquitination and promotes MHC class II survival regardless of the activation stimulus. J Biol Chem. (2010) 285:41749–54. 10.1074/jbc.M110.157586 21047782 PMC3009902

[84] Sanderson IR Ouellette AJ Carter EA Walker WA Harmatz PR. Differential regulation of B7 mRNA in enterocytes and lymphoid cells. Immunology (1993) 79:434–8. 7691725 PMC1421973

[85] Nakazawa A Watanabe M Kanai T Yajima T Yamazaki M Ogata H. Functional expression of costimulatory molecule CD86 on epithelial cells in the inflamed colonic mucosa. Gastroenterology (1999) 117:536–45. 10464129 10.1016/s0016-5085(99)70446-4

[86] Bloom S Simmons D Jewell DP. Adhesion molecules intercellular adhesion molecule-1 (ICAM-1), ICAM-3 and B7 are not expressed by epithelium in normal or inflamed colon. Clin Exp Immunol. (1995) 101:157–63. 7542573 10.1111/j.1365-2249.1995.tb02292.x PMC1553302

[87] Borcherding F Nitschke M Hundorfean G Rupp J von Smolinski D Bieber K. The CD40-CD40L pathway contributes to the proinflammatory function of intestinal epithelial cells in inflammatory bowel disease. Am J Pathol. (2010) 176:1816–27. 10.2353/ajpath.2010.090461 20133813 PMC2843472

[88] Cayabyab M Phillips JH Lanier LL. CD40 preferentially costimulates activation of CD4^+^ T lymphocytes. J Immunol. (1994) 152:1523–31. 7509825

[89] Leitner J Herndler-Brandstetter D Zlabinger GJ Grubeck-Loebenstein B Steinberger P. CD58/CD2 is the primary costimulatory pathway in human CD28-CD8^+^ T cells. J Immunol. (2015) 195:477–87. 10.4049/jimmunol.1401917 26041540

[90] Framson PE Cho DH Lee LY Hershberg RM. Polarized expression and function of the costimulatory molecule CD58 on human intestinal epithelial cells. Gastroenterology (1999) 116:1054–62. 10220497 10.1016/s0016-5085(99)70008-9

[91] Peters U Papadopoulos T Muller-Hermelink HK. MHC class II antigens on lung epithelial of human fetuses and neonates. Ontogeny and expression in lungs with histologic evidence of infection. Lab Invest. (1990) 63:38–43. 1695696

[92] Badve S Deshpande C Hua Z Logdberg L. Expression of invariant chain (CD 74) and major histocompatibility complex (MHC) class II antigens in the human fetus. J Histochem Cytochem. (2002) 50:473–82. 10.1177/002215540205000404 11897800

[93] Glanville AR Tazelaar HD Theodore J Imoto E Rouse RV Baldwin JC. The distribution of MHC class I and II antigens on bronchial epithelium. Am Rev Respir Dis. (1989) 139:330–4. 10.1164/ajrccm/139.2.330 2464294

[94] Rossi GA Sacco O Balbi B Oddera S Mattioni T Corte G. Human ciliated bronchial epithelial cells: expression of the HLA-DR antigens and of the HLA-DR alpha gene, modulation of the HLA-DR antigens by gamma-interferon and antigen-presenting function in the mixed leukocyte reaction. Am J Respir Cell Mol Biol. (1990) 3:431–9. 10.1165/ajrcmb/3.5.431 2145880

[95] Cunningham AC Milne DS Wilkes J Dark JH Tetley TD Kirby JA. Constitutive expression of MHC and adhesion molecules by alveolar epithelial cells (type II pneumocytes) isolated from human lung and comparison with immunocytochemical findings. J Cell Sci. (1994) 107:443–9. 8207072 10.1242/jcs.107.2.443

[96] Kallenberg CG Schilizzi BM Beaumont F Poppema S De Leij L The TH. Expression of class II MHC antigens on alveolar epithelium in fibrosing alveolitis. Clin Exp Immunol. (1987) 67:182–90. 3621672 PMC1542562

[97] Kaneko Y Kuwano K Kunitake R Kawasaki M Hagimoto N Hara N. B7-1, B7-2 and class II MHC molecules in idiopathic pulmonary fibrosis and bronchiolitis obliterans-organizing pneumonia. Eur Respir J. (2000) 15:49–55. 10.1034/j.1399-3003.2000.15a10.x 10678620

[98] Papi A Stanciu LA Papadopoulos NG Teran LM Holgate ST Johnston SL. Rhinovirus infection induces major histocompatibility complex class I and costimulatory molecule upregulation on respiratory epithelial cells. J Infect Dis. (2000) 181:1780–4. 10.1086/315463 10823784

[99] Tanaka H Maeda K Nakamura Y Azuma M Yanagawa H Sone S. CD40 and IFN-gamma dependent T cell activation by human bronchial epithelial cells. J Med Invest. (2001) 48:109–17. 11286011

[100] Steiniger B Sickel E. Class II MHC molecules and monocytes/macrophages in the respiratory system of conventional, germ-free and interferon-gamma-treated rats. Immunobiology (1992) 184:295–310. 10.1016/S0171-2985(11)80588-7 1592423

[101] Sacco O Lantero S Scarso L Galietta LJ Spallarossa D Silvestri M. Modulation of HLA-DR antigen and ICAM-1 molecule expression on airway epithelial cells by sodium nedocromil. Ann Allergy Asthma Immunol. (1999) 83:49–54. 10.1016/S1081-1206(10)63512-0 10437816

[102] Chang SC Hsu HK Perng RP Shiao GM Lin CY. Increased expression of MHC class II antigens in rejecting canine lung allografts. Transplantation (1990) 49:1158–63. 2113727 10.1097/00007890-199006000-00026

[103] Vignola AM Campbell AM Chanez P Bousquet J Paul-Lacoste P Michel FB. HLA-DR and ICAM-1 expression on bronchial epithelial cells in asthma and chronic bronchitis. Am Rev Respir Dis. (1993) 148:689–94. 10.1164/ajrccm/148.3.689 8103654

[104] Gao J De BP Banerjee AK. Human parainfluenza virus type 3 up-regulates major histocompatibility complex class I and II expression on respiratory epithelial cells: involvement of a STAT1- and CIITA-independent pathway. J Virol. (1999) 73:1411–8. 9882346 10.1128/jvi.73.2.1411-1418.1999 PMC103965

[105] Elssner A Jaumann F Wolf WP Schwaiblmair M Behr J Furst H. Bronchial epithelial cell B7-1 and B7-2 mRNA expression after lung transplantation: a role in allograft rejection? Eur Respir J. (2002) 20:165–9. 10.1183/09031936.02.00268102 12166565

[106] King TE. Cryptogenic Organizing Pneumonia (2017). Available online at: https://www.uptodate.com/contents/cryptogenic-organizing-pneumonia.

[107] Hershberg RM Framson PE Cho DH Lee LY Kovats S Beitz J. Intestinal epithelial cells use two distinct pathways for HLA class II antigen processing. J Clin Invest. (1997) 100:204–15. 9202073 10.1172/JCI119514 PMC508181

[108] London CA Lodge MP Abbas AK. Functional responses and costimulator dependence of memory CD4^+^ T cells. J Immunol. (2000) 164:265–72. 10.4049/jimmunol.164.1.265 10605020

[109] Croft M Bradley LM Swain SL. Naive versus memory CD4 T cell response to antigen. Memory cells are less dependent on accessory cell costimulation and can respond to many antigen-presenting cell types including resting B cells. J Immunol. (1994) 152:2675–85. 7908301

[110] Hollander D. Crohn's disease–a permeability disorder of the tight junction? Gut (1988) 29:1621–4. 10.1136/gut.29.12.1621 3065154 PMC1434087

[111] Arnott ID Kingstone K Ghosh S. Abnormal intestinal permeability predicts relapse in inactive Crohn disease. Scand J Gastroenterol. (2000) 35:1163–9. 10.1080/003655200750056637 11145287

[112] Smecuol E Bai JC Vazquez H Kogan Z Cabanne A Niveloni S. Gastrointestinal permeability in celiac disease. Gastroenterology (1997) 112:1129–36. 9097995 10.1016/s0016-5085(97)70123-9

[113] Raposo G Stoorvogel W. Extracellular vesicles: exosomes, microvesicles & friends. J Cell Biol. (2013) 200:373–83. 10.1083/jcb.201211138 23420871 PMC3575529

[114] van Niel G Raposo G Candalh C Boussac M Hershberg R Cerf-Bensussan N. Intestinal epithelial cells secrete exosome-like vesicles. Gastroenterology (2001) 121:337–49. 10.1053/gast.2001.26263 11487543

[115] Van Niel G Mallegol J Bevilacqua C Candalh C Brugiere S Tomaskovic-Crook E. Intestinal epithelial exosomes carry MHC class II/peptides able to inform the immune system in mice. Gut (2003) 52:1690–7. 10.1136/gut.52.12.1690 14633944 PMC1773888

[116] Mallegol J Van Niel G Lebreton C Lepelletier Y Candalh C Dugave C. T84-intestinal epithelial exosomes bear MHC class II/peptide complexes potentiating antigen presentation by dendritic cells. Gastroenterology (2007) 132:1866–76. 10.1053/j.gastro.2007.02.043 17484880

[117] Cunningham AC Zhang JG Moy JV Ali S Kirby JA. A comparison of the antigen-presenting capabilities of class II MHC-expressing human lung epithelial and endothelial cells. Immunology (1997) 91:458–63. 9301537 10.1046/j.1365-2567.1997.d01-2249.x PMC1364017

[118] Kalb TH Chuang MT Marom Z Mayer L. Evidence for accessory cell function by class II MHC antigen-expressing airway epithelial cells. Am J Respir Cell Mol Biol. (1991) 4:320–9. 10.1165/ajrcmb/4.4.320 2015098

[119] Suda T Sato A Sugiura W Chida K. Induction of MHC class II antigens on rat bronchial epithelial cells by interferon-gamma and its effect on antigen presentation. Lung (1995) 173:127–37. 7715254 10.1007/BF02981472

[120] Salik E Tyorkin M Mohan S George I Becker K Oei E. Antigen trafficking and accessory cell function in respiratory epithelial cells. Am J Respir Cell Mol Biol. (1999) 21:365–79. 10.1165/ajrcmb.21.3.3529 10460754

[121] Satoh A Toyota M Ikeda H Morimoto Y Akino K Mita H. Epigenetic inactivation of class II transactivator (CIITA) is associated with the absence of interferon-[gamma]-induced HLA-DR expression in colorectal and gastric cancer cells. Oncogene (2004) 23:8876–8886. 10.1038/sj.onc.1208144 15467734

[122] Cencič A Langerholc T. Functional cell models of the gut and their applications in food microbiology—a review. Int J Food Microbiol. (2010) 141:S4–14. 10.1016/j.ijfoodmicro.2010.03.026 20444515 PMC7173225

[123] Hatano R Yamada K Iwamoto T Maeda N Emoto T Shimizu M. Antigen presentation by small intestinal epithelial cells uniquely enhances IFN-gamma secretion from CD4^+^ intestinal intraepithelial lymphocytes. Biochem Biophys Res Commun. (2013) 435:592–6. 10.1016/j.bbrc.2013.05.024 23684621

[124] Thome JJ Bickham KL Ohmura Y Kubota M Matsuoka N Gordon C. Early-life compartmentalization of human T cell differentiation and regulatory function in mucosal and lymphoid tissues. Nat Med. (2016) 22:72–77. 10.1038/nm.4008 26657141 PMC4703455

[125] Maggio-Price L Seamons A Bielefeldt-Ohmann H Zeng W Brabb T Ware C. Lineage targeted MHC-II transgenic mice demonstrate the role of dendritic cells in bacterial-driven colitis. Inflamm Bowel Dis. (2013) 19:174–84. 10.1002/ibd.23000 22619032 PMC3427724

[126] Loschko J Schreiber HA Rieke GJ Esterhazy D Meredith MM Pedicord VA. Absence of MHC class II on cDCs results in microbial-dependent intestinal inflammation. J Exp Med. (2016) 213:517–34. 10.1084/jem.20160062 27001748 PMC4821651

[127] Coombes JL Siddiqui KR Arancibia-Carcamo CV Hall J Sun CM Belkaid Y. A functionally specialized population of mucosal CD103^+^ DCs induces Foxp3^+^ regulatory T cells via a TGF-β-and retinoic acid–dependent mechanism. J Exp Med. (2007) 204:1757–64. 10.1084/jem.20070590 17620361 PMC2118683

[128] Fuchs A Vermi W Lee JS Lonardi S Gilfillan S Newberry RD. Intraepithelial type 1 innate lymphoid cells are a unique subset of IL-12-and IL-15-responsive IFN-γ-producing cells. Immunity (2013) 38:769–81. 10.1016/j.immuni.2013.02.010 23453631 PMC3634355

[129] Ferrick DA Schrenzel MD Mulvania T Hsieh B. Differential production of interferon-gamma and interleukin-4 in response to Th1-and Th2-stimulating pathogens by gammadelta T cells *in vivo*. Nature (1995) 373:255. 7816142 10.1038/373255a0

[130] Vivier E Tomasello E Baratin M Walzer T Ugolini S. Functions of natural killer cells. Nat Immunol. (2008) 9:503–10. 10.1038/ni1582 18425107

[131] Skeen MJ Ziegler HK. Activation of gamma delta T cells for production of IFN-gamma is mediated by bacteria via macrophage-derived cytokines IL-1 and IL-12. J Immunol. (1995) 154:5832–41. 7538532

[132] Loh L Ivarsson M Michaelsson J Sandberg J Nixon DF. Invariant natural killer T cells developing in the human fetus accumulate and mature in the small intestine. Mucosal Immunol. (2014) 7:1233–43. 10.1038/mi.2014.13 24646938

[133] Reith W LeibundGut-Landmann S Waldburger JM. Regulation of MHC class II gene expression by the class II transactivator. Nat Rev Immunol. (2005) 5:793–806. 10.1038/nri1708 16200082

[134] Sanderson IR Bustin SA Dziennis S Paraszczuk J Stamm DS. Age and diet act through distinct isoforms of the class II transactivator gene in mouse intestinal epithelium. Gastroenterology (2004) 127:203–212. 10.1053/j.gastro.2004.04.014 15236186

[135] Ibrahim L Dominguez M Yacoub M. Primary human adult lung epithelial cells *in vitro*: response to interferon-gamma and cytomegalovirus. Immunology (1993) 79:119–24. 8099565 PMC1422047

[136] Radosevich M Ono SJ. MHC class II gene expression is not induced in HPIV3-infected respiratory epithelial cells. Immunol Res. (2004) 30:125–38. 10.1385/IR:30:2:125 15477655

[137] Mowat AM Agace WW. Regional specialization within the intestinal immune system. Nat Rev Immunol. (2014) 14:667–85. 10.1038/nri3738 25234148

[138] Jarvinen TT Collin P Rasmussen M Kyrönpalo S Mäki M Partanen J. Villous tip intraepithelial lymphocytes as markers of early-stage coeliac disease. Scand J Gastroenterol. (2004) 39:428–33. 10.1080/00365520310008773 15180179

[139] Spits H Bernink JH Lanier L. NK cells and type 1 innate lymphoid cells: partners in host defense. Nat Immunol. (2016) 17:758–64. 10.1038/ni.3482 27328005

[140] Pflanz S Timans JC Cheung J Rosales R Kanzler H Gilbert J. IL-27, a Heterodimeric cytokine composed of EBI3 and p28 protein, induces proliferation of naive CD4^+^ T cells. Immunity (2002) 16:779–90. 10.1016/S1074-7613(02)00324-2 12121660

[141] Diegelmann J Olszak T Goke B Blumberg RS Brand S. A novel role for interleukin-27 (IL-27) as mediator of intestinal epithelial barrier protection mediated via differential signal transducer and activator of transcription (STAT) protein signaling and induction of antibacterial and anti-inflammatory proteins. J Biol Chem. (2012) 287:286–98. 10.1074/jbc.M111.294355 22069308 PMC3249079

[142] Feng XM Chen XL Liu N Chen Z Zhou YL Han ZB. Interleukin-27 upregulates major histocompatibility complex class II expression in primary human endothelial cells through induction of major histocompatibility complex class II transactivator. Hum Immunol. (2007) 68:965–72. 10.1016/j.humimm.2007.10.004 18191724

[143] Hunter CA. New IL-12-family members: IL-23 and IL-27, cytokines with divergent functions. Nat Rev Immunol. (2005) 5:521–31. 10.1038/nri1648 15999093

[144] Lopetuso LR Chowdhry S Pizarro TT. Opposing functions of classic and novel IL-1 family members in gut health and disease. Front Immunol. (2013) 4:181. 10.3389/fimmu.2013.00181 23847622 PMC3705591

[145] Okamura H Nagata K Komatsu T Tanimoto T Nukata Y Tanabe F. A novel costimulatory factor for gamma interferon induction found in the livers of mice causes endotoxic shock. Infect Immun. (1995) 63:3966–72. 7558306 10.1128/iai.63.10.3966-3972.1995 PMC173557

[146] Kohno K Kataoka J Ohtsuki T Suemoto Y Okamoto I Usui M. IFN-gamma-inducing factor (IGIF) is a costimulatory factor on the activation of Th1 but not Th2 cells and exerts its effect independently of IL-12. J Immunol. (1997) 158:1541–50. 9029088

[147] Nakanishi K Yoshimoto T Tsutsui H Okamura H. Interleukin-18 is a unique cytokine that stimulates both Th1 and Th2 responses depending on its cytokine milieu. Cytokine Growth Factor Rev. (2001) 12:53–72. 10.1016/S1359-6101(00)00015-0 11312119

[148] Pizarro TT Michie MH Bentz M Woraratanadharm J Smith MF Jr. Foley E. IL-18, a novel immunoregulatory cytokine, is up-regulated in Crohn's disease: expression and localization in intestinal mucosal cells. J Immunol. (1999) 162:6829–35. 10352304

[149] Monteleone G Trapasso F Parrello T Biancone L Stella A Iuliano R. Bioactive IL-18 expression is up-regulated in Crohn's disease. J Immunol. (1999) 163:143–7. 10384110

[150] Kanai T Watanabe M Okazawa A Nakamaru K Okamoto M Naganuma M. Interleukin 18 is a potent proliferative factor for intestinal mucosal lymphocytes in Crohn's disease. Gastroenterology (2000) 119:1514–23. 10.1053/gast.2000.20260 11113073

[151] Leon AJ Garrote JA Blanco-Quiros A Calvo C Fernandez-Salazar L Del Villar A. Interleukin 18 maintains a long-standing inflammation in coeliac disease patients. Clin Exp Immunol. (2006) 146:479–85. 10.1111/j.1365-2249.2006.03239.x 17100768 PMC1810422

[152] Salcedo R Worschech A Cardone M Jones Y Gyulai Z Dai RM. MyD88-mediated signaling prevents development of adenocarcinomas of the colon: role of interleukin 18. J Exp Med. (2010) 207:1625–36. 10.1084/jem.20100199 20624890 PMC2916129

[153] Maerten P Shen C Colpaert S Liu Z Bullens DA van Assche G. Involvement of interleukin 18 in Crohn's disease: evidence from *in vitro* analysis of human gut inflammatory cells and from experimental colitis models. Clin Exp Immunol. (2004) 135:310–7. 10.1111/j.1365-2249.2004.02362.x 14738461 PMC1808939

[154] Kolinska J Lisa V Clark JA Kozakova H Zakostelecka M Khailova L. Constitutive expression of IL-18 and IL-18R in differentiated IEC-6 cells: effect of TNF-alpha and IFN-gamma treatment. J Interferon Cytokine Res. (2008) 28:287–96. 10.1089/jir.2006.0130 18547159

[155] Weiss ES Girard-Guyonvarc'h C Holzinger D de Jesus AA Tariq Z Picarsic J. Interleukin-18 diagnostically distinguishes and pathogenically promotes human and murine macrophage activation syndrome. Blood (2018) 131:1442–55. 10.1182/blood-2017-12-820852 29326099 PMC5877443

[156] Cameron LA Taha RA Tsicopoulos A Kurimoto M Olivenstein R Wallaert B. Airway epithelium expresses interleukin-18. Eur Respir J. (1999) 14:553–9. 10543274 10.1034/j.1399-3003.1999.14c12.x

[157] Muneta Y Goji N Tsuji NM Mikami O Shimoji Y Nakajima Y. Expression of interleukin-18 by porcine airway and intestinal epithelium. J Interferon Cytokine Res. (2002) 22:883–9. 10.1089/107999002760274908 12396728

[158] Wittmann M Purwar R Hartmann C Gutzmer R Werfel T. Human keratinocytes respond to interleukin-18: implication for the course of chronic inflammatory skin diseases. J Invest Dermatol. (2005) 124:1225–33. 10.1111/j.0022-202X.2005.23715.x 15955098

[159] Englyst HN Macfarlane GT Breakdown of resistant and readily digestible starch by human gut bacteria. J Sci Food Agric. (1986) 37:699–706. 10.1002/jsfa.2740370717

[160] Lorenz RG Chaplin DD McDonald KG McDonough JS Newberry RD. Isolated lymphoid follicle formation is inducible and dependent upon lymphotoxin-sufficient B lymphocytes, lymphotoxin β receptor & TNF receptor I function. J Immunol. (2003) 170:5475–82. 10.4049/jimmunol.170.11.5475 12759424

[161] Charlson ES Bittinger K Haas AR Fitzgerald AS Frank I Yadav A. Topographical continuity of bacterial populations in the healthy human respiratory tract. Am J Respir Crit Care Med. (2011) 184:957–63. 10.1164/rccm.201104-0655OC 21680950 PMC3208663

[162] Wu BG Segal LN. Lung microbiota and its impact on the mucosal immune phenotype. Microbiol Spectr. (2017) 5. 10.1128/microbiolspec.BAD-0005-2016 28643622 PMC5484071

[163] Matsumoto S Setoyama H Imaoka A Okada Y Amasaki H Suzuki K. Gamma delta TCR-bearing intraepithelial lymphocytes regulate class II major histocompatibility complex molecule expression on the mouse small intestinal epithelium. Epithelial Cell Biol. (1995) 4:163–70. 9439904

[164] Matsumoto S Nanno M Watanabe N Miyashita M Amasaki H Suzuki K. Physiological roles of gammadelta T-cell receptor intraepithelial lymphocytes in cytoproliferation and differentiation of mouse intestinal epithelial cells. Immunology (1999) 97:18–25. 10447710 10.1046/j.1365-2567.1999.00735.x PMC2326803

[165] Umesaki Y Okada Y Matsumoto S Imaoka A Setoyama H. Segmented filamentous bacteria are indigenous intestinal bacteria that activate intraepithelial lymphocytes and induce MHC class II molecules and fucosyl asialo GM1 glycolipids on the small intestinal epithelial cells in the ex-germ-free mouse. Microbiol Immunol. (1995) 39:555–62. 7494493 10.1111/j.1348-0421.1995.tb02242.x

[166] Sydora BC Mixter PF Houlden B Hershberg R Levy R Comay M. T-cell receptor gamma delta diversity and specificity of intestinal intraepithelial lymphocytes: analysis of IEL-derived hybridomas. Cell Immunol. (1993) 152:305–22. 10.1006/cimm.1993.1293 8258140

[167] Jarry A Cerf-Bensussan N Brousse N Selz F Guy-Grand D. Subsets of CD3^+^ (T cell receptor alpha/beta or gamma/delta) and CD3^−^ lymphocytes isolated from normal human gut epithelium display phenotypical features different from their counterparts in peripheral blood. Eur J Immunol. (1990) 20:1097–103. 10.1002/eji.1830200523 2141568

[168] Bolnick DI Snowberg LK Caporaso JG Lauber C Knight R Stutz WE. Major histocompatibility complex class IIb polymorphism influences gut microbiota composition and diversity. Mol Ecol. (2014) 23:4831–45. 10.1111/mec.12846 24975397

[169] Kubinak JL Stephens WZ Soto R Petersen C Chiaro T Gogokhia L. MHC variation sculpts individualized microbial communities that control susceptibility to enteric infection. Nat Commun. (2015) 6:8642. 10.1038/ncomms9642 26494419 PMC4621775

[170] Silverman M Kua L Tanca A. Protective major histocompatibility complex allele prevents type 1 diabetes by shaping the intestinal microbiota early in ontogeny. Proc Natl Acad Sci USA. (2017) 114:9671–6. 10.1073/pnas.1712280114 28831005 PMC5594701

[171] Bingula R Filaire M. Desired turbulence? Gut-lung axis, immunity, and lung cancer. J Oncol. (2017) 2017:5035371. 10.1155/2017/5035371 29075294 PMC5623803

[172] Tsay TB Yang MC Chen PH Hsu CM Chen LW. Gut flora enhance bacterial clearance in lung through toll-like receptors 4. J Biomed Sci. (2011) 18:68. 10.1186/1423-0127-18-68 21906393 PMC3179706

[173] Fagundes CT Amaral FA Vieira AT Soares AC Pinho V Nicoli JR. Transient TLR activation restores inflammatory response and ability to control pulmonary bacterial infection in germfree mice. J Immunol. (2012) 188:1411–20. 10.4049/jimmunol.1101682 22210917

[174] Yitbarek A Alkie T Taha-Abdelaziz K Astill J Rodriguez-Lecompte JC Parkinson J. Gut microbiota modulates type I interferon and antibody-mediated immune responses in chickens infected with influenza virus subtype H9N2. Benef Microbes (2018) 9:417–27. 10.3920/BM2017.0088 29380643

[175] Tweedle JL Deepe GS Jr TNFalpha antagonism reveals a gut/lung axis that amplifies regulatory T cells in a pulmonary fungal infection. Infect Immun. (2018) 86 10.1128/IAI.00109-18 PMC5964519 29581197

[176] Bradley CP Teng F Felix KM Sano T Naskar D Block KE. Segmented filamentous bacteria provoke lung autoimmunity by inducing gut-lung axis Th17 cells expressing dual TCRs. Cell Host Microbe (2017) 22:697–704 e694. 10.1016/j.chom.2017.10.007 29120746 PMC5749641

[177] Cho Y Abu-Ali G Tashiro H Kasahara DI Brown TA Brand JD. The microbiome regulates pulmonary responses to ozone in mice. Am J Respir Cell Mol Biol. (2018) 59:346–54. 10.1165/rcmb.2017-0404OC 29529379 PMC6189641

[178] Gui QF Lu HF Zhang CX Xu ZR Yang YH. Well-balanced commensal microbiota contributes to anti-cancer response in a lung cancer mouse model. Genet Mol Res. (2015) 14:5642–51. 10.4238/2015.May.25.16 26125762

[179] Lundin KEA Gjertsen HA Scott H Sollid LM Thorsby E. Function of DQ2 and DQ8 as HLA susceptibility molecules in celiac disease. Hum Immunol. (1994) 41:24–7. 10.1016/0198-8859(94)90079-5 7836060

[180] Sarna VK Skodje GI Reims HM Risnes LF Dahal-Koirala S Sollid LM. HLA-DQ:gluten tetramer test in blood gives better detection of coeliac patients than biopsy after 14-day gluten challenge. Gut (2017) 67:1606–13. 10.1136/gutjnl-2017-314461 28779027

[181] Brottveit M Raki M Bergseng E Fallang LE Simonsen B Lovik A. Assessing possible celiac disease by an HLA-DQ2-gliadin tetramer test. Am J Gastroenterol. (2011) 106:1318–24. 10.1038/ajg.2011.23 21364548

[182] Sollid LM Lie BA. Celiac disease genetics: current concepts and practical applications. Clin Gastroenterol Hepatol. (2005) 3:843–51. 10.1016/S1542-3565(05)00532-X 16234020

[183] Irvine DJ Purbhoo MA Krogsgaard M Davis MM. Direct observation of ligand recognition by T cells. Nature (2002) 419:845–9. 10.1038/nature01076 12397360

[184] Angelo M Bendall SC Finck R Hale MB Hitzman C Borowsky AD. Multiplexed ion beam imaging of human breast tumors. Nat Med. (2014) 20:436–42. 10.1038/nm.3488 24584119 PMC4110905

[185] Tang Q Henriksen KJ Boden EK Tooley AJ Ye J Subudhi SK. Cutting edge: CD28 controls peripheral homeostasis of CD4^+^CD25^+^ regulatory T cells. J Immunol. (2003) 171:3348–52. 10.4049/jimmunol.171.7.3348 14500627

[186] KleinJan A Willart MA Kuipers H Coyle AJ Hoogsteden HC Lambrecht BN. Inducible costimulator blockade prolongs airway luminal patency in a mouse model of obliterative bronchiolitis. Transplantation (2008) 86:1436–44. 10.1097/TP.0b013e3181886baa 19034015

[187] Lombardi V Singh AK Akbari O. The role of costimulatory molecules in allergic disease and asthma. Int Arch Allergy Immunol. (2010) 151:179–89. 10.1159/000242355 19786798 PMC2837887

[188] Westendorf AM Bruder D Hansen W Buer J. Intestinal epithelial antigen induces CD4^+^ T cells with regulatory phenotype in a transgenic autoimmune mouse model. Ann N Y Acad Sci. (2006) 1072:401–6. 10.1196/annals.1326.035 17057222

[189] Sato T Vries RG Snippert HJ van de Wetering M Barker N Stange DE. Single Lgr5 stem cells build crypt–villus structures *in vitro* without a mesenchymal niche. Nature (2009) 459:262–5. 10.1038/nature07935 19329995

[190] Ootani A Li X Sangiorgi E Ho QT Ueno H Toda S. Sustained *in vitro* intestinal epithelial culture within a Wnt-dependent stem cell niche. Nat Med. (2009) 15:701–6. 10.1038/nm.1951 19398967 PMC2919216

[191] McCracken KW Howell JC Wells JM Spence JR. Generating human intestinal tissue from pluripotent stem cells *in vitro*. Nat Protoc. (2011) 6:1920–8. 10.1038/nprot.2011.410 22082986 PMC3896236

[192] Rouch JD Scott A Lei NY Solorzano-Vargas RS Wang J Hanson EM. Development of functional microfold (M) cells from intestinal stem cells in primary human enteroids. PLoS ONE (2016) 11:e0148216. 10.1371/journal.pone.0148216 26820624 PMC4731053

[193] Farin HF Karthaus WR Kujala P Rakhshandehroo M Schwank G Vries RG. Paneth cell extrusion and release of antimicrobial products is directly controlled by immune cell–derived IFN-γ. J Exp Med. (2014) 211:1393–05. 10.1084/jem.20130753 24980747 PMC4076587

[194] Ettayebi K Crawford SE Murakami K Broughman JR Karandikar U Tenge VR. Replication of human noroviruses in stem cell-derived human enteroids. Science (2016) 353:1387–93. 10.1126/science.aaf5211 27562956 PMC5305121

[195] Saxena K Blutt SE Ettayebi K Zeng XL Broughman JR Crawford SE. Human intestinal enteroids: a new model to study human rotavirus infection, host restriction & pathophysiology. J Virol. (2015) 90:43–56. 10.1128/jvi.01930-15 26446608 PMC4702582

[196] Forbester JL Goulding D Vallier L Hannan N Hale C Pickard D. Interaction of salmonella enterica serovar typhimurium with intestinal organoids derived from human induced pluripotent stem cells. Infect Immun. (2015) 83:2926–34. 10.1128/iai.00161-15 25964470 PMC4468523

[197] Chen YW Huang SX de Carvalho A Ho SH Islam MN Volpi S. A three-dimensional model of human lung development and disease from pluripotent stem cells. Nat Cell Biol. (2017) 19:542–9. 10.1038/ncb3510 28436965 PMC5777163

[198] Routy B Le Chatelier E. Gut microbiome influences efficacy of PD-1-based immunotherapy against epithelial tumors. Science (2018) 359:91–7. 10.1126/science.aan3706 29097494

[199] Vetizou M Pitt JM Daillere R Lepage P Waldschmitt N Flament C. Anticancer immunotherapy by CTLA-4 blockade relies on the gut microbiota. Science (2015) 350:1079–84. 10.1126/science.aad1329 26541610 PMC4721659

[200] Van der Flier LG Clevers H. Stem cells, self-renewal & differentiation in the intestinal epithelium. Annu Rev Physiol. (2009) 71:241–60. 10.1146/annurev.physiol.010908.163145 18808327

